# Macrophage-derived exosomes promote telomere fragility and senescence in tubular epithelial cells by delivering miR-155

**DOI:** 10.1186/s12964-024-01708-5

**Published:** 2024-07-10

**Authors:** Qing Yin, Tao-Tao Tang, Xiao-Yu Lu, Wei-Jie Ni, Di Yin, Yi-Lin Zhang, Wei Jiang, Yue Zhang, Zuo-Lin Li, Yi Wen, Wei-Hua Gan, Ai-Qing Zhang, Lin-Li Lv, Bin Wang, Bi-Cheng Liu

**Affiliations:** 1 Institute of Nephrology, Zhong Da Hospital, Southeast University School of Medicine, No. 87, Dingjiaqiao Road, Nanjing, Jiangsu China; 2 Department of Pediatric Nephrology, Affiliated Maternity and Child Health Care Hospital of Nantong University, Nantong, Jiangsu China; 3 Department of Nephrology, Taixing People’s Hospital, Taixing, Jiangsu China; 4 Department of Pediatric Nephrology, The Second Affiliated Hospital of Nanjing Medical University, Nanjing, Jiangsu China; 5 Department of Pediatric Nephrology, The Fourth Affiliated Hospital of Nanjing Medical University, Nanjing, Jiangsu China

**Keywords:** CKD, Cell senescence, Macrophages, Exosomes, MiR-155, TRF1

## Abstract

**Background:**

Chronic kidney disease (CKD) is highly prevalent worldwide, and its global burden is substantial and growing. CKD displays a number of features of accelerated senescence. Tubular cell senescence is a common biological process that contributes to CKD progression. Tubulointerstitial inflammation is a driver of tubular cell senescence and a common characteristic of CKD. However, the mechanism by which the interstitial inflammation drives tubular cell senescence remains unclear. This paper aims to explore the role of exosomal miRNAs derived from macrophages in the development of tubular cell senescence.

**Methods:**

Among the identified inflammation-related miRNAs, miR-155 is considered to be one of the most important miRNAs involved in the inflammatory response. Macrophages, the primary immune cells that mediate inflammatory processes, contain a high abundance of miR-155 in their released exosomes. We assessed the potential role of miR-155 in tubular cell senescence and renal fibrosis. We subjected miR-155^−/−^ mice and wild-type controls, as well as tubular epithelial cells (TECs), to angiotensin II (AngII)-induced kidney injury. We assessed kidney function and injury using standard techniques. TECs were evaluated for cell senescence and telomere dysfunction *in vivo* and *in vitro*. Telomeres were measured by the fluorescence in situ hybridization.

**Results:**

Compared with normal controls, miR-155 was up-regulated in proximal renal tubule cells in CKD patients and mouse models of CKD. Moreover, the expression of miR-155 was positively correlated with the extent of renal fibrosis, eGFR decline and p16^INK4A^ expression. The overexpression of miR-155 exacerbated tubular senescence, evidenced by increased detection of p16^INK4A^/p21expression and senescence-associated β-galactosidase activity. Notably, miR-155 knockout attenuates renal fibrosis and tubule cell senescence *in vivo*. Interestingly, once released, macrophages-derived exosomal miR-155 was internalized by TECs, leading to telomere shortening and dysfunction through targeting TRF1. A dual-luciferase reporter assay confirmed that TRF1 was the direct target of miR-155. Thus, our study clearly demonstrates that exosomal miR-155 may mediate communication between macrophages and TECs, subsequently inducing telomere dysfunction and senescence in TECs.

**Conclusions:**

Our work suggests a new mechanism by which macrophage exosomes are involved in the development of tubule senescence and renal fibrosis, in part by delivering miR-155 to target TRF1 to promote telomere dysfunction. Our study may provide novel strategies for the treatment of AngII-induced kidney injury.

**Supplementary Information:**

The online version contains supplementary material available at 10.1186/s12964-024-01708-5.

## Background

With the development of aging of population, the prevalence of chronic kidney disease (CKD) is increasing, accounting for about 10% of the total population [1]. CKD has a high mortality and disability rate, and the burden of the disease is heavy. Currently, there is no specific treatment for CKD. Interestingly, CKD has many hallmarks of aging, such as atherosclerosis, poor wound healing, sarcopenia, infection, inflammation, weakness, skin atrophy, and cognitive impairment. Therefore, CKD can be considered as a premature aging syndrome or accelerated senescence [2].

Renal tubular epithelial cells (TECs) are the major somatic cell type in the kidney and are particularly susceptible to injury stimuli such as ischemic, hypoxic, obstructive, and metabolic insults [3, 4]. Of note, TECs are the most likely to transit to the senescent phenotype among the somatic cells of the kidney. Cell senescence is an irreversible growth arrest, which can be induced by DNA damage, over-activation of the renin–angiotensin–aldosterone system (RAAS), reactive oxygen species and persistent inflammation, all of which are commonly occurred in CKD [5, 6]. One of the most significant factors contributing to the cell senescence is chronic inflammation [7]. Studies have shown that senescent cells drive kidney fibrosis and is highly correlated with the progression of kidney damage [8–12]. However, the underlying mechanism of tubular senescence in CKD has not been well elucidated.

The primary characteristics of cellular senescence encompass telomere dysfunction, growth arrest and the accumulation of senescence-related proteins, notably p16^INK4A^ and senescence-associated-β-galactosidase (SA-β-gal) activity [4, 13–15]. Telomeres are nucleoprotein structures located at the ends of eukaryotic chromosomes, which protect them from degradation and DNA damage [16]. Studies have found that short, dysfunctional telomeres sensitize the kidneys to develop fibrosis [17, 18]. The telomere repeat-binding factor 1 (TERF1, referred to hereafter as TRF1) constitutes a crucial component of the shelterin complex localized at mammalian telomeres. It plays a pivotal role in preserving telomeric integrity by preventing telomeric fusion and fragility [19]. However, its impact on tubular senescence in CKD remains largely unknown.

MicroRNAs (miRNAs) are small non-coding RNAs that post-transcriptionally regulate gene repression. Modulating the expression of pathogenic miRNA through antisense inhibition has emerged as an exciting area for the management of various diseases [20]. Among the identified inflammation-related miRNAs, miR-155 is highly conserved one across vertebrate species and is considered to be one of the most important miRNAs involved in the inflammatory response [21]. Macrophages, the primary immune cells that mediate inflammatory processes, contain a high abundance of miR-155 in their releasing exosomes [22]. Our previous studies have demonstrated that exosomal miRNAs mediate cell–cell communication and thus play a critical role in mediating inflammation [23–27]. In addition, we found that miR-155 inhibition rescued telomeric dysfunction by enhancing TRF1 [28]. However, the impact of macrophage-derived miR-155 on tubular senescence has not been clarified.

In this study, we investigated the role of macrophage-derived exosome miR-155 in tubular senescence of CKD. Our results demonstrate that macrophage-derived exosomal miR-155 induces senescence and telomere dysfunction in proximal TECs (pTECs) via negative targeting of TRF1, providing new insights into the prevention and treatment of AngII-induced kidney injury.

## Methods

### Mice

MiR-155^−/−^ mice in a C57BL/6 background were provided by Dr. Jie Du (Beijing Anzhen Hospital, Capital Medical University, Beijing, China), which were originally purchased from the Jackson Laboratory (Bar Harbor, ME), and at 8–10 weeks old with 21–24 g body weight. C57BL/6 (wild-type) mice were aged and weight-matched (Vital River Laboratory Animal Technology Co., Ltd.). Experimental procedures were approved by the ethics committees for animal experimentation of Southeast University (No. 20191101004).

### AngII-induced chronic kidney disease

The osmotic micropump (model 2002, Alza Corporation, PaloAlto, CA) was used to be loaded with vehicle or AngII (Sigma-Aldrich, MO) in the subcutaneous tissue of the scapula to maintain a delivery rate of 0.75 μg/h for a course of 4 weeks. All the mice were also given 1% sodium chloride in high-salt drinking water. Mice were sacrificed after 4 weeks and kidney tissue was collected for various studies. Blood samples were taken before the sacrifice and transferred into eppendorf tubes. The plasma was separated by centrifugation at 3000 rpm at 4 ^◦^C for 15 min, which was collected for further analysis. Serum creatinine (SCr) was detected by a creatinine assay kit (Jiancheng). Blood urea nitrogen (BUN) was detected by an assay kit (Jiancheng).

### Morphological studies and immunohistochemistry

In brief, formalin-fixed, paraffin-embedded kidney Sects. (4-μm thick) stained with Masson’s trichrome stain and periodic acid-Schiff (PAS) stain were used to assess kidney morphology and fibrosis. The average score over 10 random segments was calculated as the tubular injury score. Immunohistochemical staining was performed using a routine protocol. The antibody is p27 (Abcam Cat# ab92741). All immunohistochemical analyses were repeated at least 3 times and representative images are shown.

### Cell culture, transfection, and cell treatment

Mouse TECs (mTECs; a gift from J. B. Kopp, National Institutes of Health) were cultured in DMEM/F12 (Gibco, USA) supplemented with 10% FBS in a 37 °C incubator with 5% CO_2_. A mouse macrophage cell line RAW264.7 (ATCC) was cultured in DMEM medium, high glucose (Gibco, USA) supplemented with 10% FBS. The media was changed on days 2 and 5, and then every 3 days. MiR-155 inhibitors, miR-155 mimics and negative control (NC) were designed and synthesized by GenePharma (Shanghai, China). TRF1 siRNA were designed and synthesized by Hanbio (Shanghai, China). The sequences of miR-155 inhibitors and corresponding controls were 5'-AACCCCUAUCACGAUUAGCAUUAA-3' and 5'- UCUACUCUUUCUAGGAGGUUGUGA-3', respectively. The sequences of TRF1 siRNA, and NC were 5'-CCAAAUUCUCAUAUGCCUUTT-3', and 5 '- UUCUCCGAACGUGUCACGUTT-3, respectively. mTECs were transfected with lipofectamine 3000 (Invitrogen) according to the manufacturer's protocol. In short, cells were seeded in 6-well plates (2–3 × 10^5^ cells/well) and cultured to 60%-80% confluence. The transfection complex was prepared according to the manufacturer's instructions and added directly to the cells. The final concentration of TRF1 siRNA, miR-155 inhibitor, miR-155 mimic and NC was 100 nM. Then 1 × 10^–6^ μM/L of AngII was added after transfection for 48 h.

### Western blotting

A 100 × protease inhibitor was added with RIPA lysis buffer (Servicebio) to extract total proteins from kidney and cells, and SDS-PAGE isolated 4% to 20%. The proteins were then transferred to PVDF membranes (Millipore) and were blocked in NcmBlot blocking buffer (NCM Biotech) at room temperature for 10–15 min. Then membranes were incubated overnight with primary antibodies as follows: anti-TRF1 (Abcam Cat# ab1423, RRID:AB_301006), anti-γH2AX (Abcam Cat# ab26350), anti-p27 (Abcam Cat# ab92741), anti-p16^INK4A^ (Cell Signaling Technology Cat# 80772), anti-Fibronectin (Sigma-Aldrich Cat# F3648), anti-α-SMA (Abcam Cat# ab5694), anti-Alix (Santa Cruz Biotechnology Cat# sc-53540) and anti-CD9 (Santa Cruz Biotechnology Cat# sc-13118). Secondary antibodies were used for detection by an ECL advanced system (GE Healthcare). Intensity values expressed as the relative protein expression were normalized to GAPDH (ab2000, Abways). The gray bands were analyzed with ImageJ software (NIH, Bethesda, MD, USA) to compare the expression between targeted proteins and internal controls.

### Immunofluorescence staining

Immunofluorescence analysis was performed on 4 μm thick renal tissue sections and mTEC cells. They were performed with anti-TRF1 (Abcam Cat# ab1423), anti-γH2AX (Abcam Cat# ab26350), anti-p16^INK4A^ (Santa Cruz Biotechnology Cat# sc-1661), Fibronectin (Sigma-Aldrich Cat# F3648), anti-α-SMA (Abcam Cat# ab5694), anti-CD63 (GB11620, Servicebio), anti-CD68 (GB113109, Servicebio), Anti-AQP1 (Abcam Cat# ab9566), DiO (C1038, Beyotime Biotechnology) and incubated with secondary antibodies. In confocal microscope, 10 fields of view were randomly assigned and was evaluated blind. Human biopsy sections were obtained from diagnostic renal biopsies performed at the Zhong Da Hospital, Southeast University School of Medicine, and stained with anti-γH2AX (Abcam Cat# ab26350), anti-p16^INK4A^ (Santa Cruz Biotechnology Cat# sc-1661) and miR-155 FISH. All the studies involving human kidney sections were approved by the institutional ethics committee of the Zhong Da Hospital. An informed consent form was signed by all patients in accordance with the Declaration of Helsinki, and the study was approved by the Zhong Da Hospital, Southeast University (Approval number: 2017ZDSYLL107-Y02).

### Dual-luciferase reporter assay

mTECs were co-transfected with 3’-UTR-specific luciferase reporter constructs, miRNA (miRNA-NC or miR-155-5p), and Renilla luciferase using lipofectamine 3000 (Invitrogen). After 48 h of transfection, the luciferase activity of cells was measured by a Dual Luciferase Assay Kit (Promega) and microplate reader (Tecan M1000).

### Quantitative real-time PCR

Total RNA from the kidney tissues, cells, or exosomes were extracted using RNAiso (Takara). mRNA was quantified using qRT-PCR with SYBR Green (Takara). Mature miRNAs were quantified with the qRT-PCR detection kit using miRNA specific primers (GeneCopoeia). qRT-PCR was performed using the 7300 Real-Time PCR System (Applied Biosystems, Foster City, CA).

### Telomere Q-FISH analysis

Quantitative telomere fluorescence in situ hybridization (Q-FISH) directly on kidney sections was performed as previously described [29]. Telomere software was used to quantify the fluorescence intensity of telomeres. The analysis was conducted blind by two different researchers and 10 random images were analyzed. Results were presented in terms of telomere fluorescence intensity.

Telomeric dysfunction-induced lesions (TIFs) – To determine the presence of telomeric DNA damage signals, the telomeres were labeled with Cy3-labeled PNA telomere probes (F1002, Panagene), followed by immunofluorescence staining for γH2AX. γH2AX immunostaining was performed with γH2AX antibody (Abcam Cat# ab26350) overnight, followed by a secondary antibody. TIFs were quantitated manually by measuring the co-location of telomere probes and γH2AX lesions.

### Fluorescence in situ hybridization (FISH)

Cy3-labeled probe sequences of miR-155-5p were devised by Genepharma (Shanghai, China). 4-μm formalin-fixed, paraffin-embedded kidney sections were digested with protease K (20 μg/mL) and prehybridized in pre-hybridization buffer for 30 min (37 °C), followed by hybridization using Cy3-labeled miR-155-5p probes overnight (37 °C). The sections were then washed with saline-sodium citrate buffer at 42 °C to remove the unhybridized probe. The FISH images were captured under confocal microscopy.

### SA–β-gal staining

The Senescence β-galactosidase Staining Kit (Cell Signaling Technology, #9860) was used in primary mouse tubular cells and 7-μm frozen kidney tissue sections to evaluate β-galactosidase activity at pH 6, a known characteristic of senescence, according to the manufacturer's instructions.

### Exosome purification and characterization

RAW 264.7 was cultured in DMEM medium, high glucose (Gibco, USA) without serum when the medium was collected to purify exosomes. Supernatant fractions collected from ctrl cell cultures and cells treated with AngII for 48 h were subjected to exosome extraction. All samples were centrifuged at 2000 × g for 20 min to eliminate the cells and debris and at 13,500 × g for 30 min, followed by ultracentrifugation at 200,000 × g for 120 min (Type 70 Ti rotor, Beckman Coulter Optima L-80 XP). The exosome pellet was washed in 20 mL of PBS and collected by ultracentrifugation at 200,000 × g for 120 min. The washed pellets were reconstituted in 200 μL PBS and stored at − 80 °C for further analysis. The size distribution, morphology, and quantity were detected by electron microscopy and nanoparticle tracking analysis (NTA). Protein concentrations and surface markers of isolated exosomes were detected by western blotting.

### Isolation and tracking of labeled exosomes

RAW 264.7 was cultured in serum free medium for 24 h. Then cells were stained by DiO dye (5 mg/mL, Beyotime, China) for 30 min at 37 °C and the free dye was washed away with PBS. Labeled exosomes were isolated from cell supernatants using ultracentrifugation and applied to the mTEC cells that were grown in exosome-free media. After 48 h, the uptake of the labeled exosome was visualized by a confocal microscope using a 488 nm laser.

### Statistical analysis

Data was expressed as mean ± standard deviation (SD). The comparison between the two groups was performed using a two-tailed unpaired Student's t-test. One-way ANOVA was used to compare three or more groups, followed by Bonferroni correction for multiple comparisons. All analyses were performed using SPSS 22.0. A *P* value of < 0.05 is considered significant.

## Results

### MiR-155 is upregulated in proximal tubular cells (pTECs) and is associated with tubular senescence in CKD

**Fig. 1:**
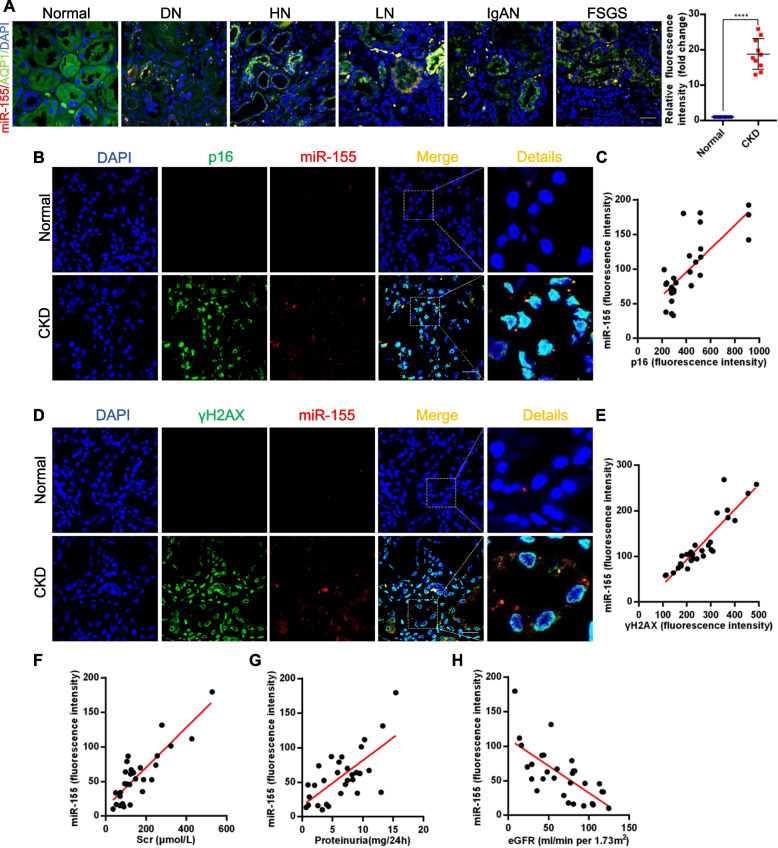
miR-155 is increased in multiple types of clinical nephropathy and is associated with tubular senescence. **A** Representative images show the expression and localization of miR-155 in various types of human CKD. miR-155 (Red), AQP1(Green). Scale bar, 50 μm. **B** Co-staining of p16^INK4A^ (green) and miR-155 (red) in various types of human CKD. **C** Scatter plots with linear regression show significant correlation between miR-155 expression levels and p16^INK4A^. *r* = 0.7115, *P* < 0.05, *n* = 30, Scale bar, 50 μm. **D** Co-staining of γH2AX (green) and miR-155 (red) in various types of human CKD. **E** Scatter plots with linear regression show significant correlation between miR-155 expression levels and γH2AX. *r* = 0.9105, *P* < 0.05, *n* = 30, Scale bar, 50 μm. **F** Linear regression shows an positive correlation between miR-155 expression level and serum creatinine levels (*r* = 0.7794, *P* < 0.05, *n* = 30), **G** proteinuria (*r* = 0.6191, *P* < 0.05, *n* = 30) and **H** an inverse correlation between miR-155 expression level and eGFR (*r* = -0.7130, *P* < 0.05, *n* = 30). DN: diabetic nephropathy; HN: hypertensive nephropathy; LN: lupus nephritis; IgAN: IgA nephropathy; FSGS: focal segmental glomerulosclerosis; CKD: chronic kidney disease

To identify the role of miR-155 in the pathogenesis of CKD, we performed miR-155 FISH on kidney biopsy specimens from 30 patients with various nephropathies, including diabetic nephropathy, hypertensive nephropathy, lupus nephritis, IgA nephropathy, and focal segmental glomerulosclerosis. As shown in Fig. 1A, miR-155 was significantly upregulated in all biopsy specimens from CKD patients compared to normal kidneys. Notably, miR-155 FISH co-staining of immunofluorescence of AQP1, a marker of pTEC, showed that elevated miR-155 was mainly located in pTECs in kidneys (Fig. 1A). To clarify the correlation between miR-155 and cellular senescence, we assessed the expression of the p16^INK4A^ (a typical senescence-related protein marker) and miR-155 FISH by immunofluorescence. Interestingly, it was found that the expression of miR-155 was nearly completely co-localized with p16^INK4A^-positive tubules (Fig. 1B and C). γH2AX is a DNA damage marker and a hallmark of cellular senescence. We found that miR-155 FISH was also co-localized with γH2AX (Fig. 1D and E). Furthermore, the correlation analysis indicated that miR-155 was positively correlated with proteinuria, serum creatinine and decline of eGFR (Fig. 1F-H). Demographic and clinical data for CKD patients are presented in Table S1. These results suggest that miR-155 is highly involved in renal tubule cell senescence and is associated with kidney injury in CKD.

### MiR-155 exacerbates cellular senescence in tubular cells *in vitro*

**Fig. 2:**
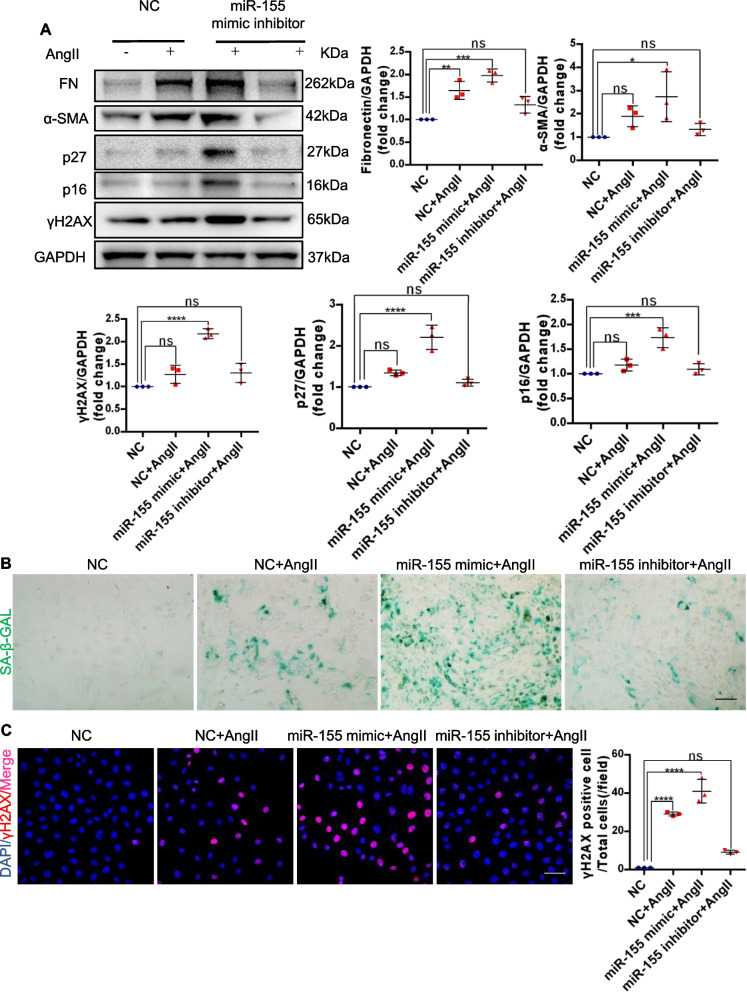
Transfection of miR-155 into cultured mouse tubular cells (mTECs) exacerbates cellular senescence *in vitro*. **A** Representative western blotting and summarized data of fibronectin, ɑ-SMA, p27, p16^INK4A^ and γH2AX in the mTECs (*n* = 3). **B** Representative images of SA-β-gal activity staining of mTECs. Scale bars, 50 µm. **C** Representative images of γH2AX stained sections of mTECs. Data are presented as mean ± SD, * *p* < 0.05, ** *p* < 0.01, *** *p* < 0.001, **** *p* < 0.0001

We then examined the effects of miR-155 *in vitro* on cellular senescence. The cultured mTECs were transfected with a miR-155 mimic and inhibitor. We adopted AngII, a major fibrogenic factor, to treat mTECs. mTECs were pretreated with miR-155 mimic/inhibitor/NC for 6 h, then incubated with AngII for 48 h. RT-PCR was used to detect the level of miR-155 in each group (Fig. S1). As shown in Fig. 2A, miR-155 significantly induced the expression of fibrosis-related proteins fibronectin and α–smooth muscle actin (α-SMA). We next examined other senescence-related proteins p16^INK4A^ and p27, and found miR-155 mimics could significantly up-regulated the expression of senescence-related proteins (Fig. 2A). Moreover, incubation with miR-155 mimics triggered the upregulation of SA–β-gal activity (Fig. 2B). The staining also showed that miR-155 mimics markedly induced a significant increase of γH2AX in the mTECs (Fig. 2C). We also detected the expression of fibrosis-related proteins and found that miR-155 mimics significantly exacerbated the expression of fibronectin and α-SMA. Consistently, treatment with miR-155 inhibitors reversed these changes in AngII induced mTECs (Fig. 2A-C). Meanwhile, consistent results were also observed *in vitro*, as shown by RT-PCR detection of fibronectin, α-SMA, p16^INK4A^, p27 and γH2AX (Fig. S2). Therefore, these results suggesting that miR-155 plays an important role in tubular senescence and exacerbates the fibrogenic response.

### Knockout of miR-155 attenuates renal fibrosis and senescence *in vivo*

**Fig. 3:**
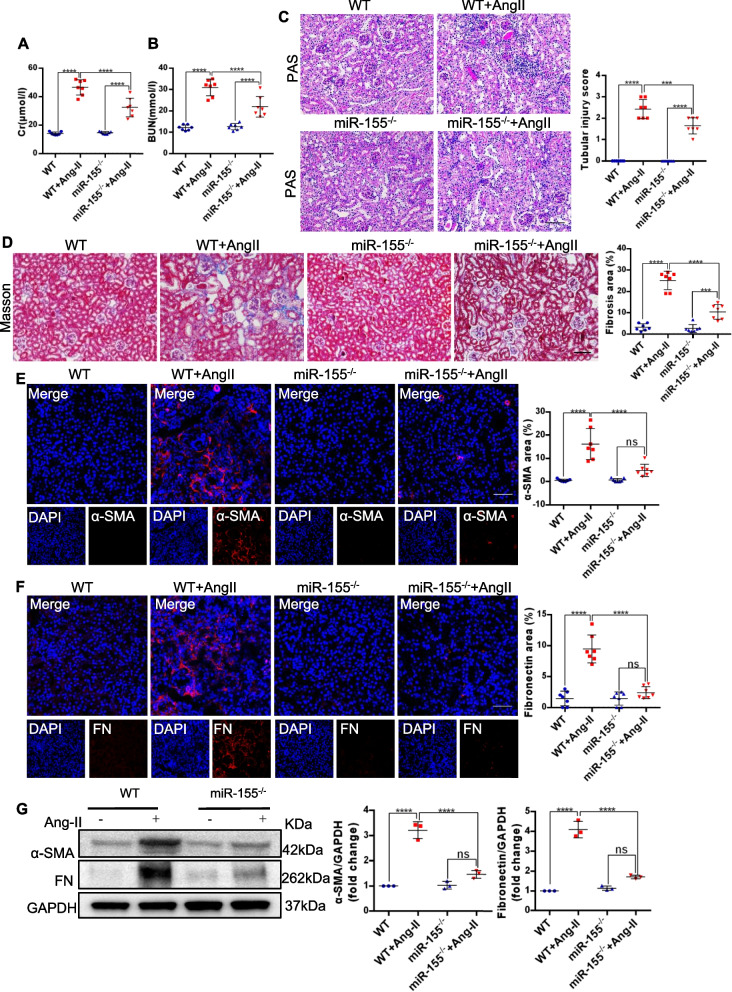
MiR-155 knockout attenuates renal fibrosis in AngII-induced kidney injury. **A** Serum creatinine levels and **B** BUN over time 4 weeks after vehicle or AngII infusion (*n* = 7). **C** Representative kidney histology as shown by PAS staining. Dot plot shows the corresponding quantification of tubular injury score (*n* = 7). Scale bar: 100 μm. **D** Representative images of masson staining of the kidney. Scale bar: 100 μm. The dot plot showed the corresponding quantification of fibrosis area (*n* = 7). **E** Representative images of ɑ-SMA-stained kidney sections from wild type and miR-155^−/−^ mice. Scale bars: 50 μm. The quantification of ɑ-SMA fluorescence intensity in the kidneys (*n* = 7). **F** Representative images of fibronectin-stained kidney sections from wild type and miR-155^−/−^ mice. Scale bars: 50 μm. The quantification of fibronectin fluorescence intensity in the kidneys (*n* = 7). **G** Representative western blotting gel documents and summarized data showing the protein levels of ɑ-SMA and fibronectin in the kidneys of mice. Data are presented as mean ± SD, * *p* < 0.05, ** *p* < 0.01, *** *p* < 0.001, **** *p* < 0.0001

To explore the potential role of miR-155 in tubular senescence, we subjected miR-155^−/−^ mice and wild-type (WT) mice, to AngII-induced kidney injury. Our results showed that miR-155 expression was significantly increased in AngII induced kidney (Fig. S3 A and B). As shown in Fig. 3A and B, serum creatinine (Scr) and blood urea nitrogen (BUN) were increased in AngII mice. However, these effects were suppressed by miR-155 knockout. Periodic acid–Schiff (PAS) staining was performed to assess damaged tubules. It was found that AngII treatment induced tubular injury in WT mice, but this was alleviated by miR-155 knockout (Fig. 3C). We further investigated the effect of miR-155 on renal fibrotic lesions after AngII treatment. Masson trichrome staining and immunofluorescence staining of fibronectin and α-SMA indicated that *in vivo* miR-155 knockout attenuated renal fibrosis after AngII treatment. Western blotting results for fibronectin and α-SMA were consistent with these staining results (Fig. 3D-G).

**Fig. 4:**
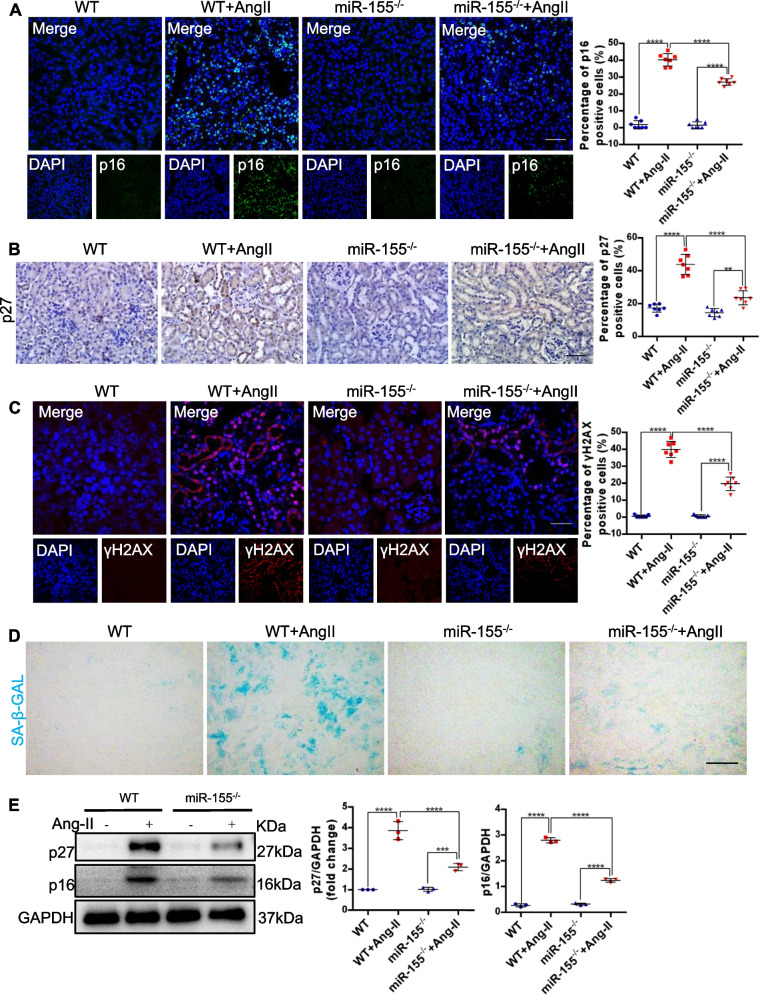
MiR-155 knockout attenuates renal TECs senescence in AngII-induced kidney injury. **A** Representative images of p16^INK4A^ staining in kidney tissues and quantification of the p16^INK4A^ positive cells (*n* = 7). Scale bars, 50 µm. **B** Representative images of p27 staining in kidney tissues and quantification of the p27 positive cells (*n* = 7). Scale bars, 50 µm. **C** Representative images of γH2AX-stained kidney sections. Scale bars: 50 μm. **D** SA-β-gal activity staining of frozen kidney sections, which appeared as bright blue granular staining in the cytoplasm of renal TECs. Scale bars, 50 µm. **E** Representative western blotting gel documents and summarized data showing the protein levels of p16^INK4A^ and p27 in the kidneys of mice. Data are presented as mean ± SD, * *p* < 0.05, ** *p* < 0.01, *** *p* < 0.001, **** *p* < 0.0001

To further clarify whether miR-155 promotes renal fibrosis by aggravating cellular senescence, we evaluated the level of tubular senescence. As shown in Fig. 4A-C, AngII-induced increase in p16^INK4A^, p27 and γH2AX were greatly blocked by miR-155 knockout. In addition, the staining analyses revealed that miR-155 knockout largely blocked the AngII-induced increase in SA–β-gal activity in tubules (Fig. 4D). Western blotting results for p16^INK4A^, p27 and γH2AX proteins showed similar results (Fig. 4E). Taken together, Our data suggest the important role of miR-155 in the tubular senescence and the development of renal fibrosis, and that miR-155 knockout attenuates these responses in AngII-induced kidney injuries.

### Knockout of miR-155 protects against telomere dysfunction in pTECs by enhancing TRF1

**Fig. 5:**
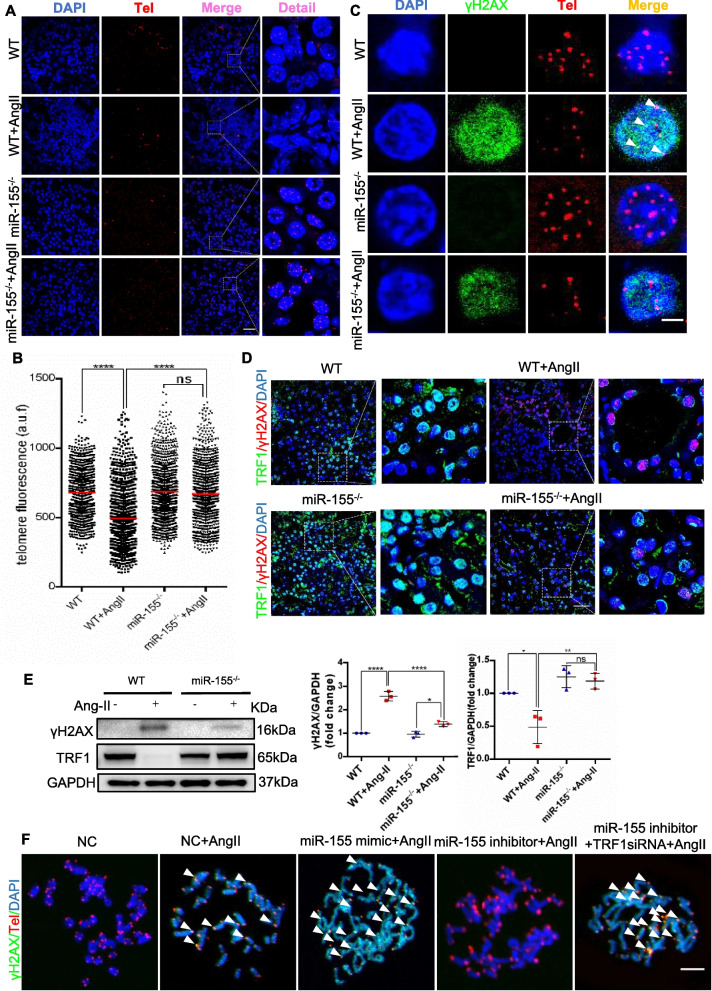
miR-155 inhibition protects telomere function by binding to TRF1 in renal epithelial cells. **A** Representative images of telomere-stained kidney sections from wild type and AngII mice 4 weeks after vector or AngII infusion. Scale bars: 50 μm. **B** Telomeric length measurements by quantitative telomeric DNA FISH in the kidney sections; a.f.u, arbitrary fluorescence units. **C** Telomeric DNA damage was detected by the co-localization of γH2AX (green) and telomeres (red) in the immunofluorescence of the kidney sections. **D** Representative images of TRF1- (green) and γH2AX- (red) stained sections of kidney sections from mice. Scale bars: 50 μm. **E** Representative western blotting and summarized data of TRF1 and γH2AX in kidney tissues of mice (*n* = 3). **F** Telomere DNA damage in mTECs treated with AngII, as demonstrated by the co-localization of γH2AX (green) and telomeres (red) in the immunofluorescence. White arrows indicate TIFs. Scale bars: 10 μm. TIFs, telomere dysfunction induced foci. Data are presented as mean ± SD, * *p* < 0.05, ** *p* < 0.01, *** *p* < 0.001, **** *p* < 0.0001

Our previous study found that miR-155 was involved in TECs telomeric DNA damage by regulating TRF1 in a cisplatin-induced AKI model [28]. However, the role of miR-155 and TRF1 in CKD conditions remains unclear. To further explore the underlying mechanisms of miR-155 in tubular senescence and renal fibrosis, we investigated miR-155/TRF1/ telomere dysfunction in AngII-induced kidney injury. In the first place, we measured telomere length by using quantitative FISH (Q-FISH). As shown in Fig. 5A and B, the telomere fluorescence signal intensity is significantly reduced in WT mice, which was reversed in miR-155^−/−^ mice. Previous studies have shown that γH2AX is associated with extremely short/dysfunctional telomeres, and its colocalization with telomere is known as telomeric dysfunction-induced lesions (TIFs) [30]. Here a dramatic increase of γH2AX was found in the kidneys of AngII-induced WT mice, indicating the strong DNA damage activation (Fig. 5C and D). We performed γH2AX immunofluorescence and telomere DNA FISH, and found more TIFs in AngII-induced WT mice compared with miR-155^−/−^ mice (Fig. 5C). Moreover, enhanced TRF1 levels by miR-155 knockout attenuated AngII-induced DNA damage in pTECs (Fig. 5D). Western blotting results for γH2AX and TRF1 proteins showed similar results (Fig. 5E).

Interestingly, we found that miR-155 mimics increased telomere fragility and telomere sister chromatid fusions, whereas miR-155 inhibition resulted in a significant suppression of these responses in AngII treated mTEC cells (Fig. 5F). Increased telomere fragility and sister chromatid fusions was aggravated by TRF1 inhibition in mTEC cells (Fig. 5F). Therefore, miR-155 contributes to telomere DNA damage after AngII infusion, and miR-155 inhibition protects against AngII-induced telomere fragility and chromosome fusion in TECs by enhancing TRF1. Furthermore, a dual-luciferase reporter assay confirmed that TRF1 was indeed a direct target of miR-155 (Fig. S4). Taken together, these data demonstrated that miR-155 knockout protects against telomere dysfunction in TECs by enhancing TRF1.

### MiR-155 inhibition protects against cell senescence and renal fibrosis induced by AngII *in vitro* by binding to TRF1

**Fig. 6:**
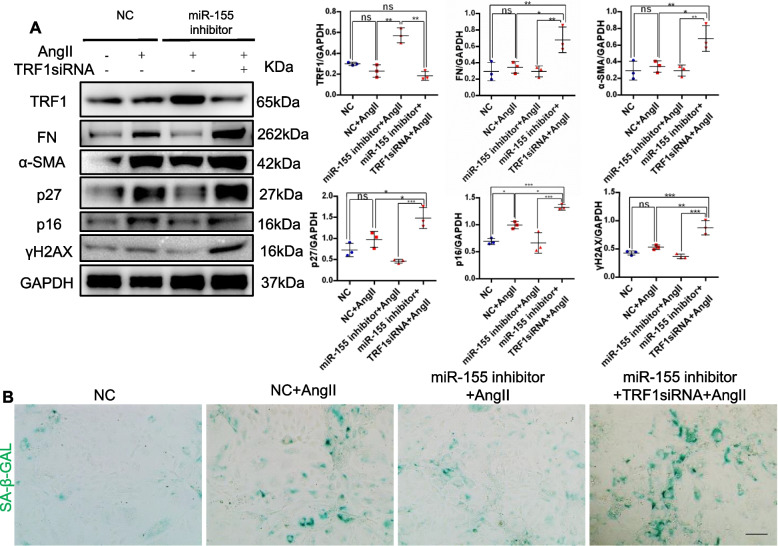
miR-155 inhibition protects cell senescence and renal fibrosis in mTECs by binding to TRF1. **A** Representative western blotting and summarized data of TRF1, fibronectin, ɑ-SMA, p27, p16^INK4A ^and γH2AX in the mTECs (*n* = 3). **B** Representative images of SA-β-gal activity staining of mTECs. Scale bars, 50 µm. Data are presented as mean ± SD, * *p* < 0.05, ** *p* < 0.01, *** *p* < 0.001, **** *p* < 0.0001

To further confirm the role of miR-155/TRF1 in senescence and fibrosis in pTECs, mTECs were transfected with miR-155 inhibitor or TRF1 siRNA. Our previous study has shown that miR-155 inhibitor/TRF1 siRNA transfection alone does not affect cell phenotype [28].Treatment of AngII induced increased expression of fibronectin and α-SMA, both of which were blocked by miR-155 inhibition (Fig. 6A). Moreover, the siRNA-induced TRF1 knock-down blocked protective effects of miR-155 inhibitor in cell senescence and fibrotic lesions after AngII treatment (Fig. 6A and B). These findings suggest that miR-155 inhibition reduces cell senescence and fibrotic lesions in mTECs by binding to TRF1.

### Macrophage-derived exosomes transfer miR-155 into tubules in AngII-induced kidney

Interestingly, the level of TRF1 in kidneys was significantly reduced after AngII intervention *in vivo* (Fig. 5E), while there was no significant change in cultured mTECs after AngII treatment *in vitro* (Fig. 6A), indicating that miR-155 that has an effect on TRF1 may not originate from the tubular cells themselves. MiR-155 was found to be induced in macrophages and macrophage-derived exosomal miR-155 mediated cardiomyocyte apoptosis and uremic cardiomyopathy in uremic mice [24], which stimulated us to link this with TECs senescence.

**Fig. 7:**
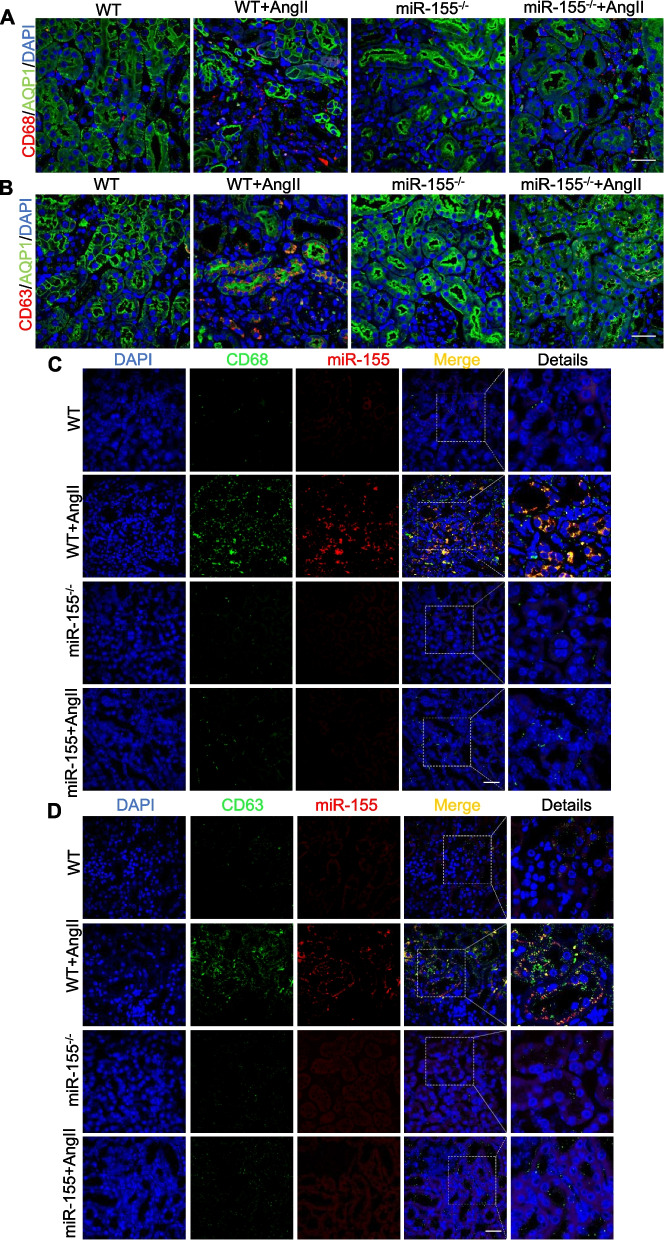
Macrophage-derived exosomes transfers miR-155 into tubules in AngII-induced kidney. **A** Representative images of AQP1- (green) and CD68 (red) stained sections of the kidney sections. Scale bars: 50 μm. **B** Representative images of AQP1- (green) and CD63 (red) stained sections of the kidney sections. Scale bars: 50 μm. **C** Representative images of miR-155 FISH (red) co-stained with CD68 (green) immunofluorescence. Scale bars: 50 μm. **D** Representative images of miR-155 FISH (red) co-stained with CD63 (green) immunofluorescence. Scale bars: 50 μm

To this end, we first performed immunostaining of CD68 (a marker of macrophage) and CD63 (a marker of exosome) to verify the presence of increased macrophages infiltration and exosomes in tubular cells in AngII-induced WT mice, whereas miR-155 knockout reversed these effects(Fig. 7A and B). To prove that macrophage-derived exosomes could be transferred into TECs, we did co-immunofluorescence of CD68 and CD63 and showed that macrophage derived exosomes could be transferred to pTECs (Fig. S5). Immunofluorescencerevealed that miR-155 is mainly expressed in the pTECs (Fig. 1A and Fig. S3). We next used miR-155 FISH combined with CD68 immunofluorescence to assess whether miR-155 is derived from macrophages. As expected, miR-155 and CD68 were co-localized in tubular with a dot-like shape (Fig. 7C). Furthermore, miR-155 also co-located CD63 *in vivo*, as evidenced by co-staining of miR-155 FISH and CD63 immunofluorescence (Fig. 7D). Overall, these data suggest that macrophage-derived exosomes transfer miR-155 into pTECs in AngII-induced kidney injury.

### Macrophage-derived exosomal miR-155 worsens telomere fragility, chromosomal instability and senescence in AngII-induced mTECs

**Fig. 8:**
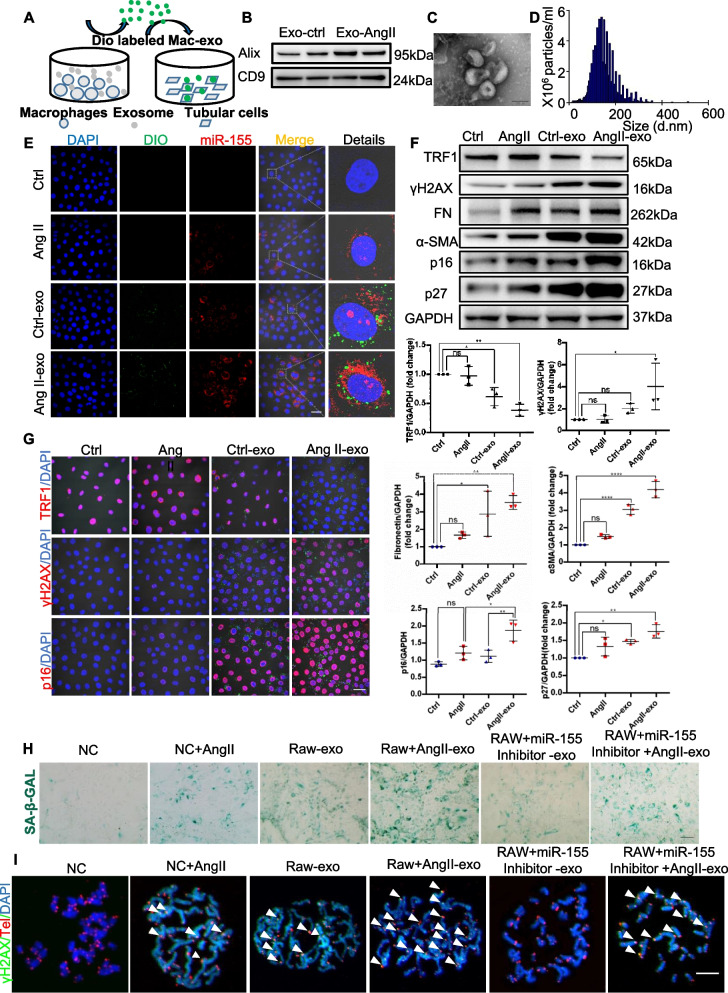
Macrophage-derived exosomes transfers miR-155 into tubules in AngII-induced kidney. **A** Exosomes harvested from the medium of RAW 264.7 were labeled with DIO and added to primary cultured mouse renal tubular cells for 48 h. **B** Western blotting for Alix and CD9 exosome markers. **C** and **D** Macrophage original exosome was verified by transmission electron microscopy, ranging in size from 80 to 130 nm. **E** DIO-labeled exosomes were introduced into mTECs for 48 h, and miR-155FISH was performed. Scale bars: 20 μm. **F** Western blotting analysis of TRF1, γH2AX, Fibronectin, α-SMA, p16^INK4A^ and p27 in primary cultured mouse renal tubular cells. (*n* = 3). **G** Representative confocal images of TRF1, γH2AX and p16^INK4A^ staining in kidney tissues. **H** Representative images of SA-β-gal activity staining of primary cultured mouse renal tubular cells. Scale bars, 50 µm. **I** Telomere DNA damage in mTECs treated with AngII, as demonstrated by the co-localization of γH2AX (green) and telomeres (red) in the immunofluorescence. White arrows indicate TIFs. Scale bars: 10 μm. TIFs, telomere dysfunction induced foci. Data are presented as mean ± SD, * *p* < 0.05, ** *p* < 0.01, *** *p* < 0.001, **** *p* < 0.0001

To further verify our hypothesis *in vitro*, dio-labeled exosomes (Fig. 8A), verified by western blotting of Alix and CD9 (Fig. 8B) and transmission electron microscopy to have a size ranging from 80–130 nm (Fig. 8C and D), were transferred into mTECs. To further clarify whether macrophage-derived exosomes increase telomere fragility, chromosomal instability and senescence through miR-155, we stimulated mTECs with AngII in the presence or absence of macrophage-derived exosomes. As expected, it was found that miR-155 was significantly increased in mTECs after the treatment of macrophage-derived exosomes (Fig. 8E). Interestingly, macrophage-derived exosomal miR-155 worsened cell senescence, telomere fragility and chromosome instability in mTECs stimulated with AngII, as evidenced by the increased expression of fibronectin, ɑ-SMA, p16^INK4A^, p27, γH2AX, SA-β-gal positive cells and TIFs(Fig. 8F-I). Furthermore, macrophage-derived exosomal miR-155 decreased TRF1 (Fig. 8F and G). However, all of the changes were attenuated by the treatment with miR-155 inhibitors (Fig. 8H and I, Fig. S6), consistently demonstrated that macrophage-derived exosomal miR-155 involved in pTECs senescence.

## Discussion

**Fig. 9:**
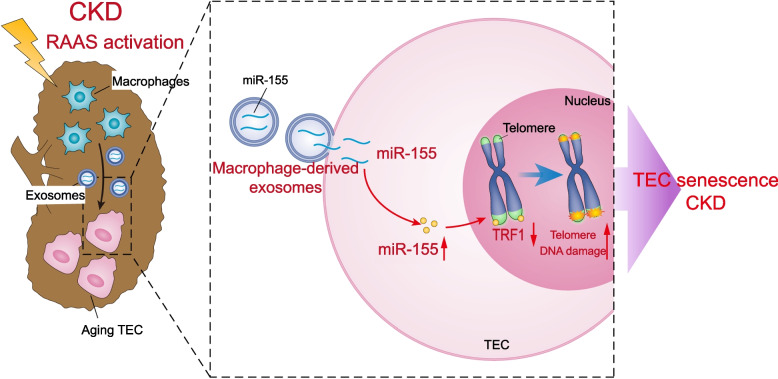
Schematic illustration of the mechanism by which macrophage-derived exosomes deliver miR-155 to promote telomere fragility, chromosomal instability, and senescence in TECs. MiR-155 was synthesized and loaded into the exosomes in increased infiltration of macrophages in AngII-infused mice. The released exosomal fusion with the plasma membrane leads to the release of miR-155 into the TECs and the translational repression of TRF1 in the TECs. Finally, macrophage-derived miR-155-containing exosomes were shown to promoted TEC tubular senescence and renal fibrosis by directly targeting TRF1 in AngII-infused mice

Recent studies have suggested that the cellular senescence contributes to the progression of renal fibrosis in CKD, however, the underlying mechanism is largely unknown. In this study, we demonstrated that macrophage-derived exosomes encapsulated with miR-155 could be taken up by pTECs and promote telomere shortening and dysfunction, followed by cellular senescence and renal fibrosis. More importantly, we proposed that the exosome/miR-155/TRF1 axis may play a crucial role in this pathological process. Our findings may provide a novel insight on TECs senescence in the progression of CKD (Fig. 9).

Tubulointerstitial inflammation is a common pathological feature of CKD, which triggers the evolution of interstitial fibrosis [31, 32]. Compared to the general population, patients with CKD also exhibit the characteristics of a premature senescence syndrome, including many of the hallmarks of aging, such as atherosclerosis, poor wound healing, sarcopenia, weakness, cognitive impairment, increased persistent inflammation and fibrosis [2, 10]. Accelerated senescence has been observed even in patients with normal GFR in proteinuria, suggesting that cellular senescence is an early event in CKD [33, 34]. TECs are the largest number of cells in the kidney and are particularly vulnerable to ischemia, hypoxia, toxins and metabolic damage. Among the various resident cells in the kidney, TECs are frequently implicated in renal senescence. Senescent cells not only lose their ability to grow and repair, but also secrete senescence associated secretory phenotype (SASP) components, including proinflammatory cytokines and growth factors that attract inflammatory cells and affect neighboring cells [35, 36]. Ttherefore, persistent inflammation, premature aging and CKD perhaps share common regulatory patterns of distinct biological pathways. However, the mechanism between inflammation and cellular senescence in TECs remains unclear.

Telomeres are the genomic portions at the ends of linear chromosomes that protect them from DNA damage. Telomere shortening and DNA damage are recognized causes of cellular senescence and aging [13, 37]. TRF1 has been implicated in telomere length regulation and telomere fusion [38–40]. However, the effect of TRF1 in tubular senescence under CKD stress is not yet understood. Since the over-activation of RAAS is a major feature of CKD progression, we therefore established the *in vivo* and *in vitro* model of AngII induced kidney injury. Notably, TRF1 was significantly down-regulated in the kidneys of AngII-induced mice (Fig. 5E). However, there was no significant change in TRF1 levels in cultured mTECs after AngII treatment, suggesting that the reduction in TRF1 might not be a direct effect of AngII. Previously, we found that knockout of miR-155 could ameliorate cisplatin-induced telomere DNA damage by enhancing TRF1 [28]. Of note, TRF1 was also identified as a direct target of miR-155 in mice with CKD (Fig. S4). Moreover, inhibition of miR-155 prevents telomere shorting and sister chromatid fusion by enhancing TRF1, preventing senescence in tubular cells(Fig. 5). Consistently, mice with deletion of the TRF1 spontaneously develop renal fibrosis, although the mechanism is unclear [17]. Therefore, the data presented here provide compelling evidence that miR-155/TRF1 is responsible for short, dysfunctional telomeres and plays a role in renal fibrosis.

Although expression of miR-155 was not per se increased in AngII treated TECs *in vitro*, we did observe a significant increase in miR-155 in tubular cells *in vivo* (Fig. 1A and Fig. S3). This motivates us to consider where miR-155 came from *in vivo*. Exosomes are nanosized membrane macrovesicles that have the capability to mediate intercellular communication by their components (i.e., miRNA, mRNA, proteins, and DNA) [32, 33]. Previous studies have found that exosomal miR-19b-3p mediates the communication between injured TECs and macrophages, and plays an important pathological role in tubulointerstitial inflammation [25]. Similar findings have also been demonstrated in other disease models [23–27]. It is well known that cellular senescence is caused not only by the pathogenic factors of the cell itself, but also by the cellular environment and its surrounding cells [32, 33]. Previously, Lanna et al. found that exosomes released by antigen-presenting cells inhibited T cell senescence by delivering telomeres [41]. Interestingly, our study found that there was increasingly co-localized expression of CD68 (macrophages marker) and CD63 (exosome marker) in tubular cells, and so do miR-155 and CD68 in AngII-induced mice (Fig. 7 and Fig. S5). Then we demonstrated that exosomal miR-155 derived from macrophages could significantly increase telomere dysfunction and senescence by down regulating TRF1 in AngII treated TECs (F ig. 8I). It should be noted that the cargos of exosomes are complex, and whether other cargos in macrophage exosomes may also contribute to TEC senescence requires further investigation. In addition, AngII treatment alone could not induce changes in miR-155 and TRF1 levels *in vitro*, which might induce cell senescence through other mechanisms. In summary, our data clearly suggested exosomal miR-155 derived from macrophages may be a key mediator of TECs senescence in AngII-induced kidneys.

Accumulating evidence suggested that tubular cell senescence contributed to progressive CKD, however, the mechanism of tubular cell senescence was not fully understood, especially the relationship between cellular senescence and inflammation. Exosome-mediated miRNA transfer is an essential mechanism of intercellular communication [42]. Recently, it has been reported that exosomes can transfer cargos between cells to mediate communication during cell senescence [32, 34]. Our study clearly suggests that infiltrating inflammatory cells, such as macrophages, may contribute to renal fibrosis bymediating tubular cell senescence through miR-155 during CKD progression. Although only AngII-induced renal injury model was used in the *in vitro* and *in vivo* experiments here, a correlation between miR-155 and cellular senescence has also been observed in the CKD patients with different etiologies. Moreover, chronic inflammation is a common feature of CKD, suggesting that our findings have universal implications in CKD. Thus, in the case of CKD, macrophages not only secrete cytokines and initiate inflammatory signals [24], but also promote tubular senescence through the transfer of miR-155 by exosomes, thereby amplifying the inflammatory responses and aggravating fibrosis. Inhibition of exosome release may be a novel target for anti-inflammatory therapy of CKD by preventing cell senescence.

## Conclusions

In summary, the present study identifies a novel mechanism by which macrophage exosomes participate in the development of tubule senescence and renal fibrosis, in part by delivering miR-155 targeting TRF1 to promote telomere dysfunction. Our study may provide novel strategies for the treatment of AngII-induced kidney injury.

### Supplementary Information

Supplementary Material 1.

Supplementary Material 2.

## Data Availability

No datasets were generated or analysed during the current study.

## Abbreviations

BUN: Blood urea nitrogen
CKD: Chronic kidney disease
mTECs: Mouse TECs
NC: Negative control
PAS: Periodic acid-Schiff
Q-FISH: Quantitative telomere fluorescence in situ hybridization
RAAS: Renin–angiotensin–aldosterone system
SA-β-gal: Senescence-associated-β-galactosidase
SASP: Senescence associated secretory phenotype
SCr: Serum creatinine
SD: Standard deviation
TECs: Tubular epithelial cells
TIFs: Telomeric dysfunction-induced lesions
TRF1: Telomere repeat-binding factor 1
