# Supplementary material for: Macrophage-derived exosomes promote telomere fragility and senescence in tubular epithelial cells by delivering miR-155
Source: Cell Commun Signal. 2024 Jul 10;22:357. doi: 10.1186/s12964-024-01708-5 (PMC11238407; doi:10.1186/s12964-024-01708-5)
Supplement: Supplementary file 1 — Supplementary Material 1. [file 12964_2024_1708_MOESM1_ESM.docx]

**Table S1. Demographic and clinical data for CKD patients.**

| **Patient**  **#** | **Gender** | **Age** | **Diagnosis** | **Serum creatinine**  **(umol/L)** | **Proteinuria**  **（mg/24h）** | **miR-155**  **(fluorescence intensity)** |
| --- | --- | --- | --- | --- | --- | --- |
| 1 | M | 22 | LN | 278 | 13.28 | 131.28 |
| 2 | M | 31 | IgAN | 107 | 0.92 | 46.35 |
| 3 | M | 42 | HN | 111 | 6.53 | 86.79 |
| 4 | F | 46 | IgAN | 72 | 9.10 | 34.25 |
| 5 | M | 42 | DN | 148 | 8.34 | 54.02 |
| 6 | F | 70 | FSGS | 171 | 7.45 | 70.05 |
| 7 | M | 66 | DN | 137 | 9.50 | 62.72 |
| 8 | M | 69 | FSGS | 105 | 6.06 | 79.18 |
| 9 | M | 31 | HN | 528 | 15.45 | 179.64 |
| 10 | F | 39 | LN | 94 | 7.14 | 46.86 |
| 11 | M | 37 | DN | 187 | 7.47 | 52.88 |
| 12 | M | 33 | IgAN | 118 | 2.03 | 45.73 |
| 13 | F | 19 | FSGS | 49 | 6.35 | 33.74 |
| 14 | M | 48 | DN | 123 | 8.23 | 60.86 |
| 15 | M | 64 | DN | 248 | 2.73 | 73.74 |
| 16 | M | 52 | DN | 182 | 13.00 | 35.55 |
| 17 | M | 36 | DN | 255 | 4.80 | 87.25 |
| 18 | M | 57 | DN | 228 | 3.57 | 52.69 |
| 19 | M | 42 | DN | 133 | 8.92 | 64.06 |
| 20 | F | 51 | DN | 323 | 9.74 | 101.28 |
| 21 | F | 71 | DN | 71 | 1.16 | 28.73 |
| 22 | F | 45 | FSGS | 427 | 10.28 | 111.86 |
| 23 | M | 29 | FSGS | 121 | 2.59 | 16.24 |
| 24 | M | 56 | FSGS | 98 | 5.82 | 64.02 |
| 25 | F | 50 | FSGS | 53 | 1.00 | 16.79 |
| 26 | M | 53 | FSGS | 90 | 0.65 | 13.75 |
| 27 | F | 16 | LN | 36 | 3.32 | 10.24 |
| 28 | F | 20 | LN | 126 | 11.02 | 67.05 |
| 29 | F | 31 | LN | 71 | 4.33 | 15.10 |
| 30 | F | 34 | LN | 83 | 4.01 | 17.95 |
| 31 | F | 48 | RC | 72 | 0.08 | 17.43 |
| 32 | M | 43 | RC | 85 | 0.07 | 25.47 |
| 33 | F | 24 | KI | 98 | 0.31 | 58.35 |
| 34 | F | 55 | RC | 57 | 0.28 | 36.27 |
| 35 | M | 36 | KI | 84 | 0.06 | 37.40 |
| 36 | M | 69 | RC | 76 | 0.32 | 20.17 |

M: Male; F: Female; DN: diabetic nephropathy; HN: hypertensive nephropathy; LN: lupus nephritis; IgAN: IgA nephropathy; FSGS: focal segmental glomerulosclerosis; RC: Renal Carcinoma; KI: Kidney injury from a traffic accident.

**Fig. S1**

**

**

Fig. S1. Angiotensin II treatment alone could not induce changes in miR-155. Real-time PCR analysis of miR-155 levels (n = 3).

**Fig. S2**

**
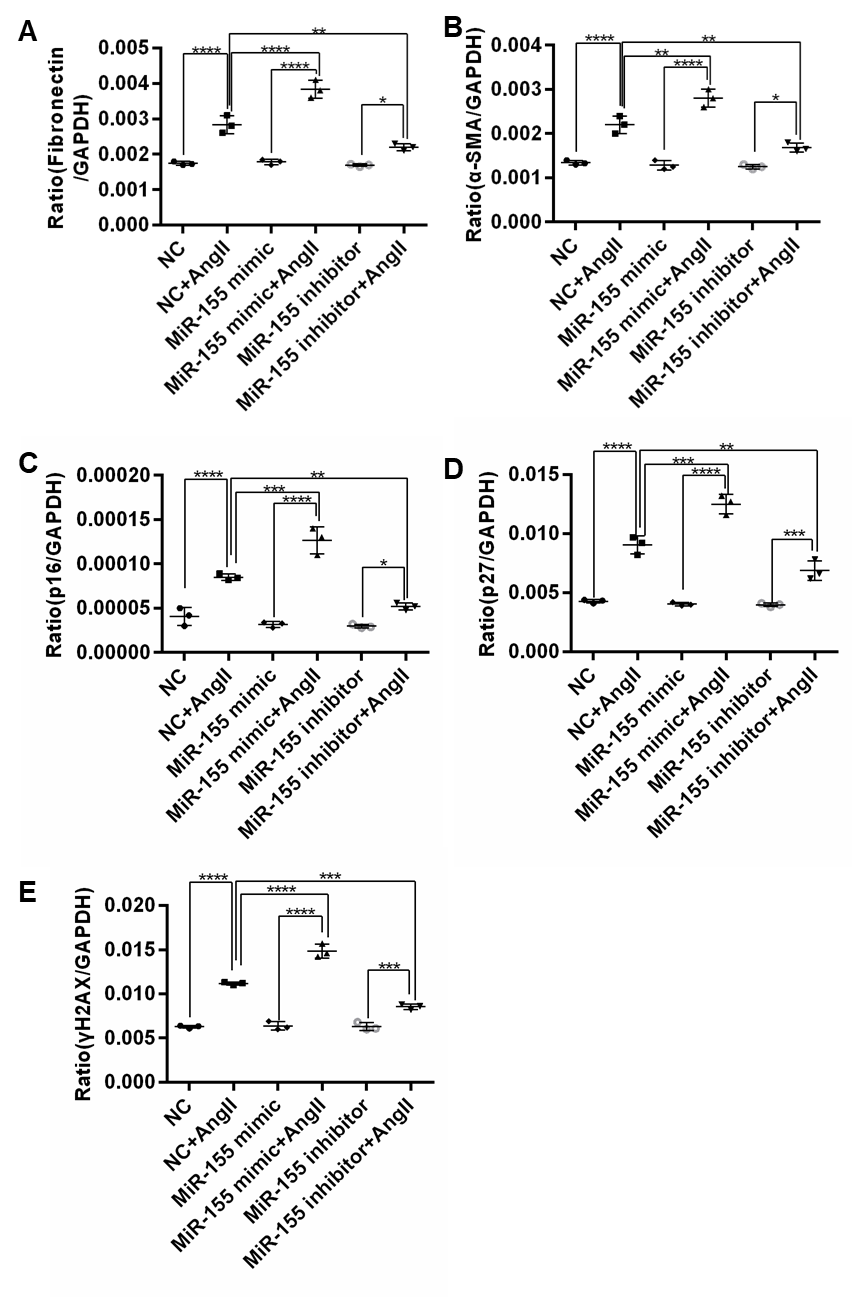
**

Fig. S2. Transfection of miR-155 into mTECs exacerbates cellular senescence in vitro. Real-time PCR analysis of fibronectin, α-SMA, p16^INK4A^, p27 and γH2AX mRNA levels (n = 3). Data are presented as mean ± SD, * p < 0.05, ** p < 0.01, *** p < 0.001, **** p < 0.0001.

**Fig. S
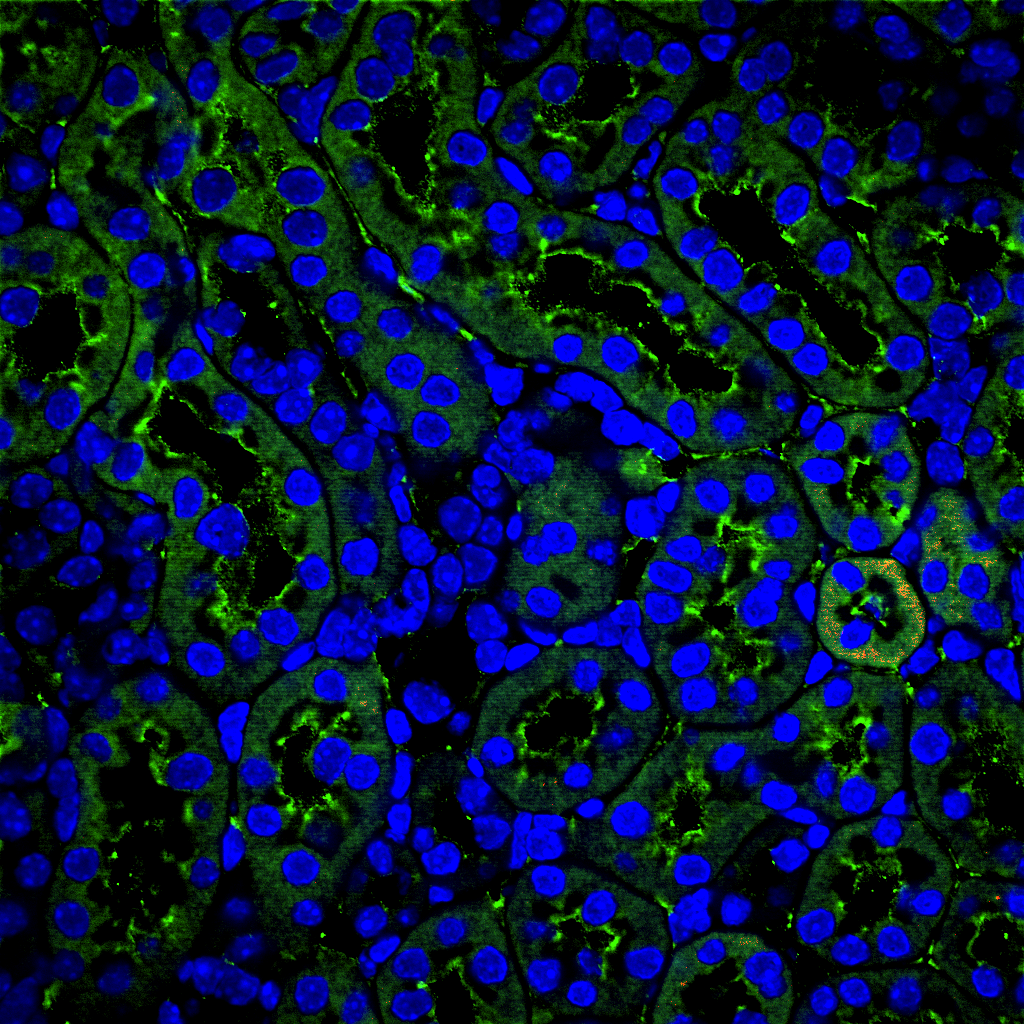
3**

**WT+AngII**

**WT**

**DAPI/miR-155/AQP1**

**DAPI/miR-155/AQP1**

**A**




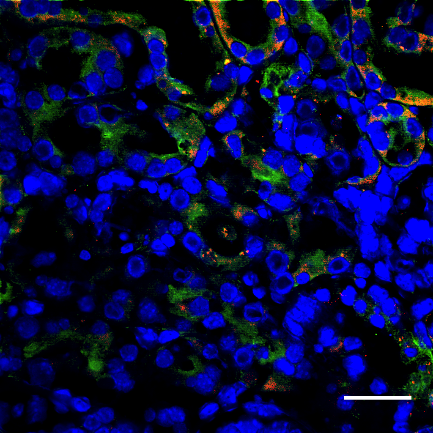


**B**

**Fig. S3. miR-155 was increased in the kidneys of AngII-induced mice.** (A) Representative images show the expression and localization of miR-155 in the kidneys of AngII-induced mice. miR-155 (Red), AQP1(Green). Scale bar, 50 μm. (B) Real-time PCR analysis of miR-155 levels in wild type and mice kidneys after Ang II administration for 4 weeks. n=3; Data are presented as mean ± SD, * p < 0.05, ** p < 0.01, *** p < 0.001, **** p < 0.0001.

**Fig. S4**


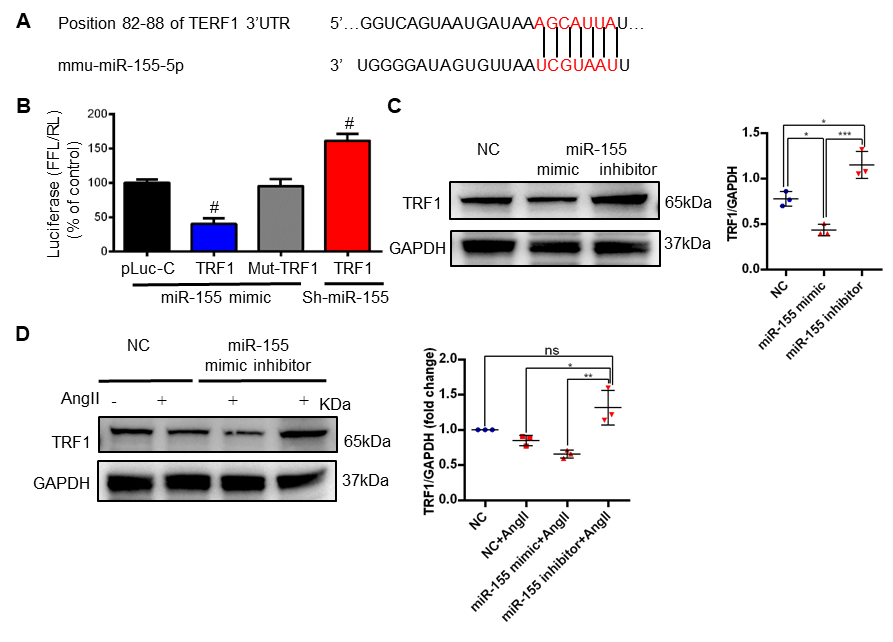


**Fig. S4** **TRF1 is a direct downstream target of miR-155.** (A) The schematic diagram depicted the predicted binding site of mmu-miR-155 targeting the 3’-UTR of TRF1. (B) Luciferase reporter assay determined TRF1 as a bona fide target of miR-155 (n = 3). (C) Representative western blotting and summarized data of TRF in mTECs (n = 3). (D) Representative western blotting and summarized data of TRF in mTECs (n = 3). Data are presented as mean ± SD, * p < 0.05, ** p < 0.01, *** p < 0.001, **** p < 0.0001.

**Fig. S5**


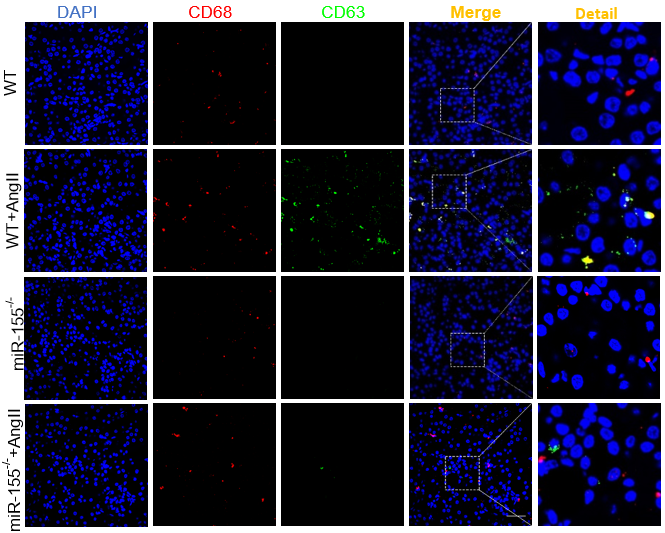


**Fig. S5. Macrophage-derived exosomes are transferred into tubule cells.** Representative images of CD63-(green) and CD68 (red) stained sections of the kidney sections. Scale bars: 50 μm.

**Fig. S6.**

Raw+miR-155 inhibitor-exo+angII












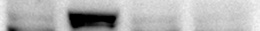

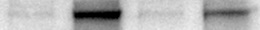

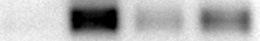

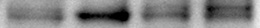

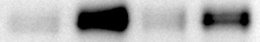

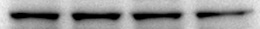


Raw+miR-155 inhibitor-exo

Raw-exo+AngII

Raw-exo

FN

p27

α-SMA

GAPDH

p16

γH2AX

262kDa

42kDa

27kDa

37kDa

16kDa

16kDa

**Fig. S6. MiR-155 inhibitors alleviate cell senescence and fibrotic states promoted by macrophage-derived exosomes in mTECs.** Western blotting analysis of γH2AX, Fibronectin, α-SMA, p16INK4A and p27 in primary cultured mouse tubular cells (n = 3). Data are presented as mean ± SD, * p < 0.05, ** p < 0.01, *** p < 0.001, **** p < 0.0001.
