# Supplementary material for: Macrophage-derived exosomes promote telomere fragility and senescence in tubular epithelial cells by delivering miR-155
Source: Cell Commun Signal. 2024 Jul 10;22:357. doi: 10.1186/s12964-024-01708-5 (PMC11238407; doi:10.1186/s12964-024-01708-5)

Unedited images for figure 2A

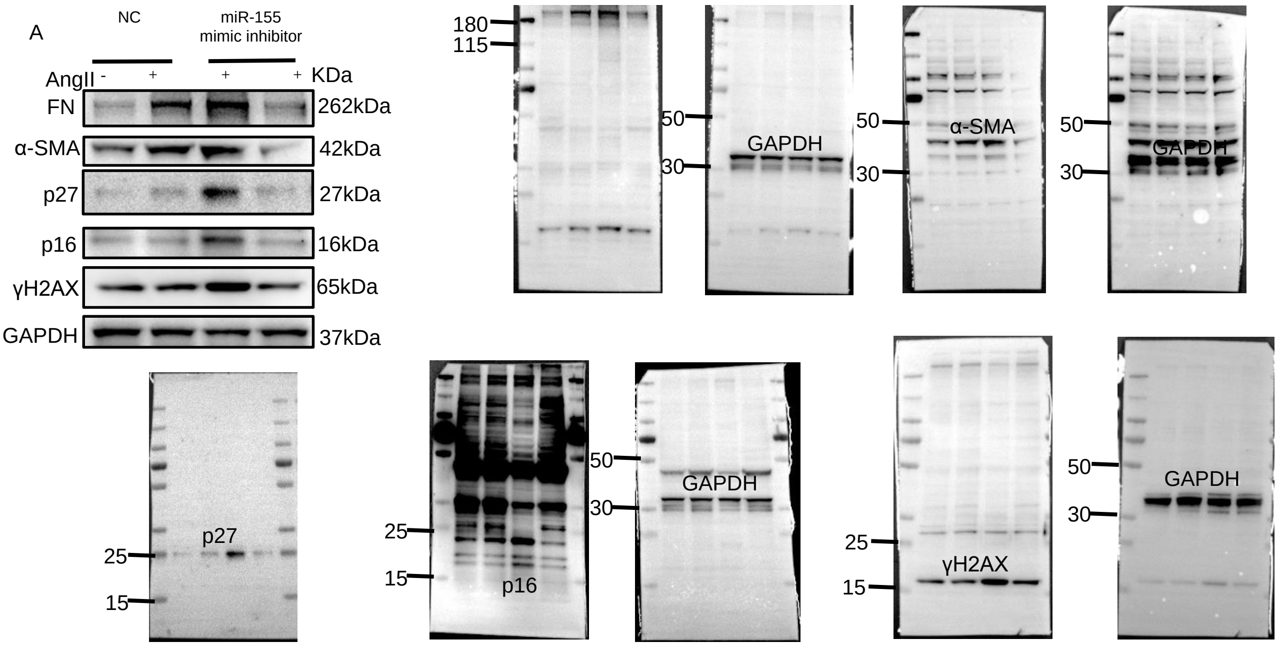

Unedited images for figure 3G

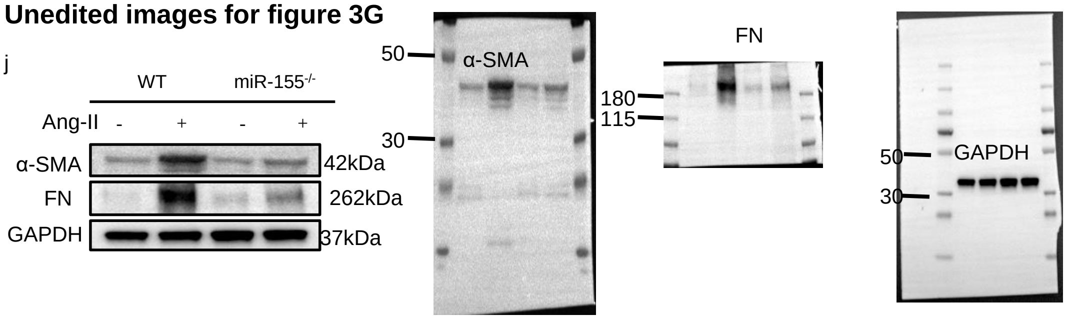

Unedited images for figure 4E

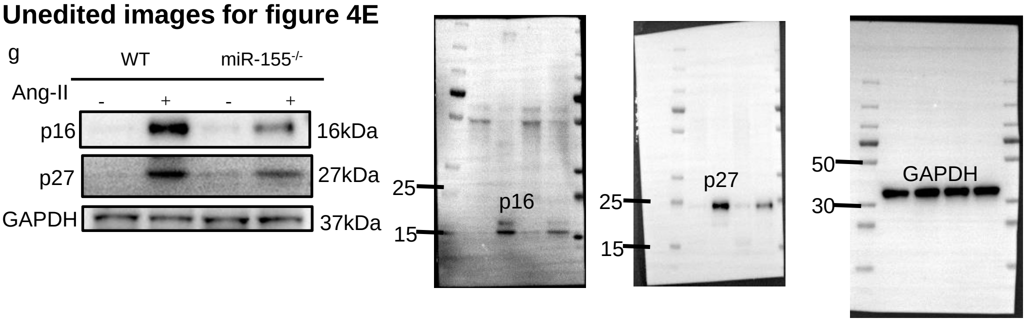

Unedited images for figure 5E

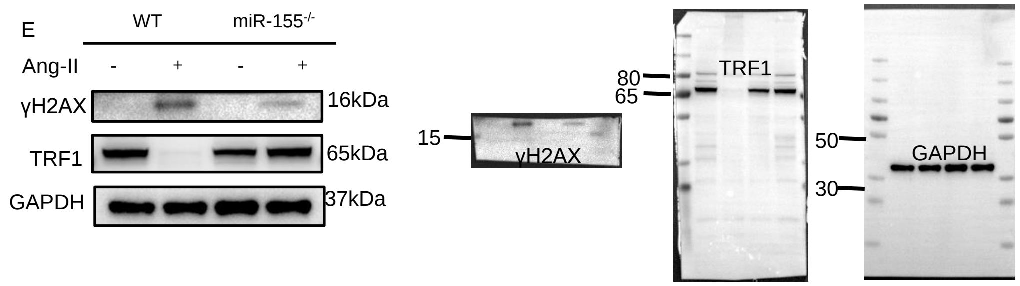

Unedited images for Figure 6A

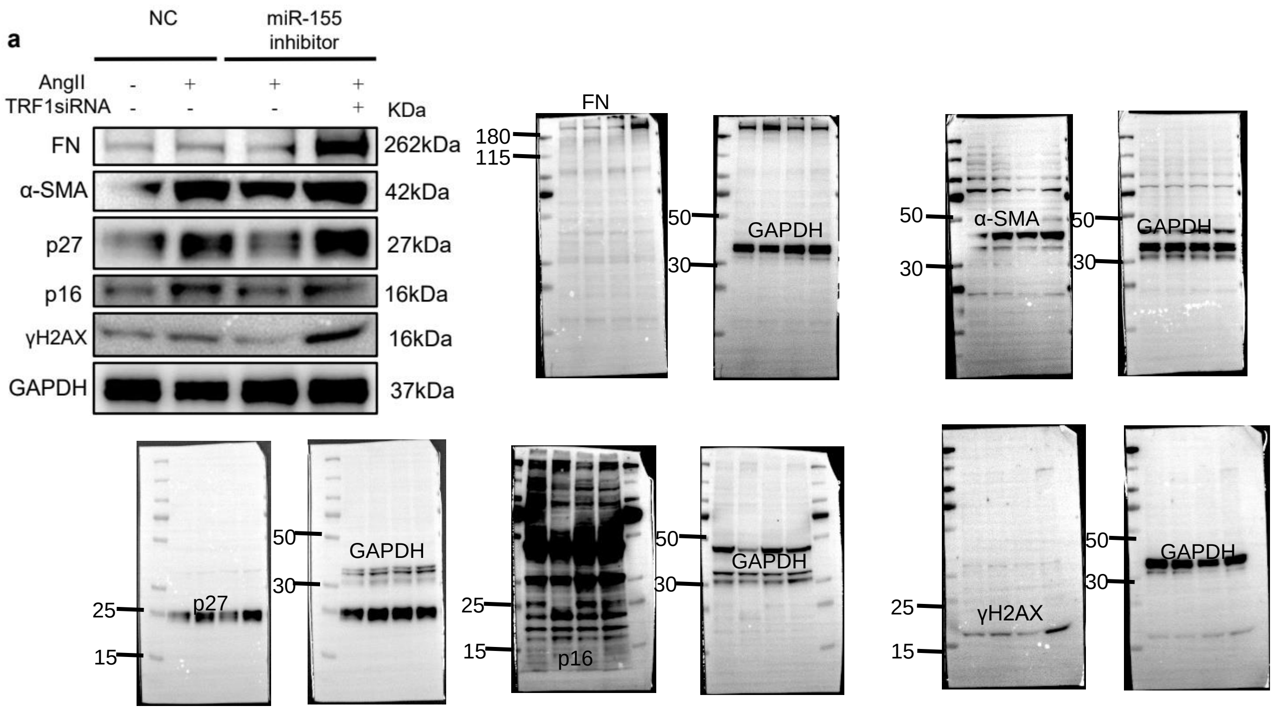

Unedited images for figure 8B

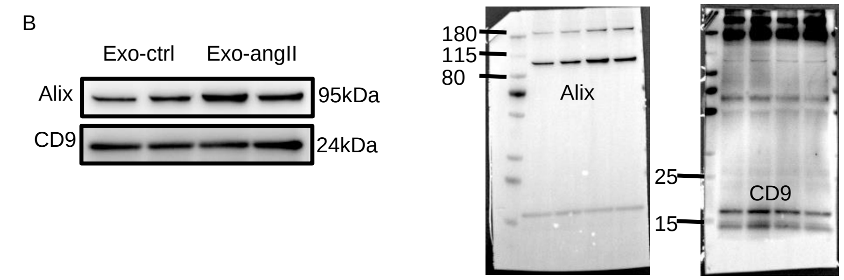

Unedited images for figure 8F

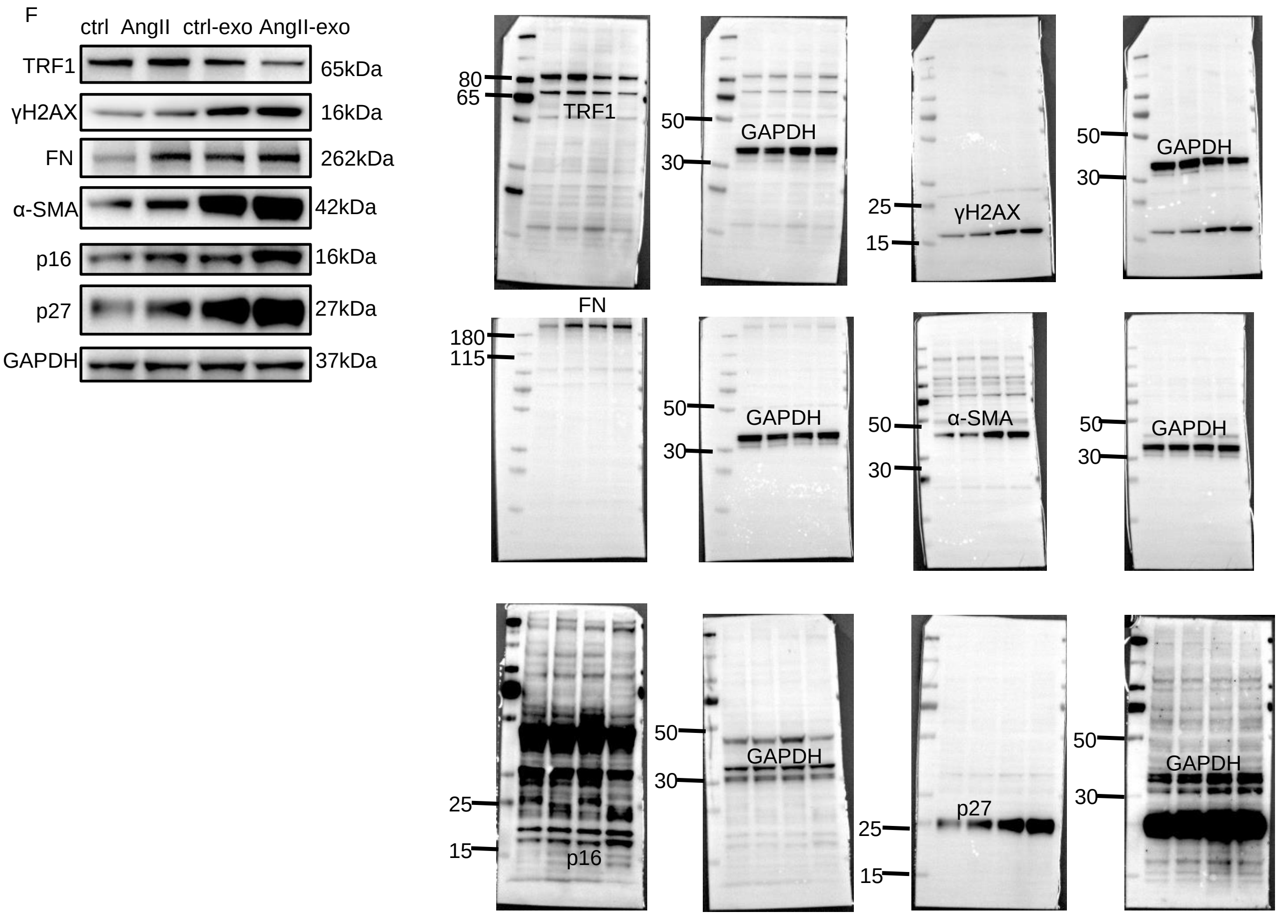

Unedited images for Figure S3

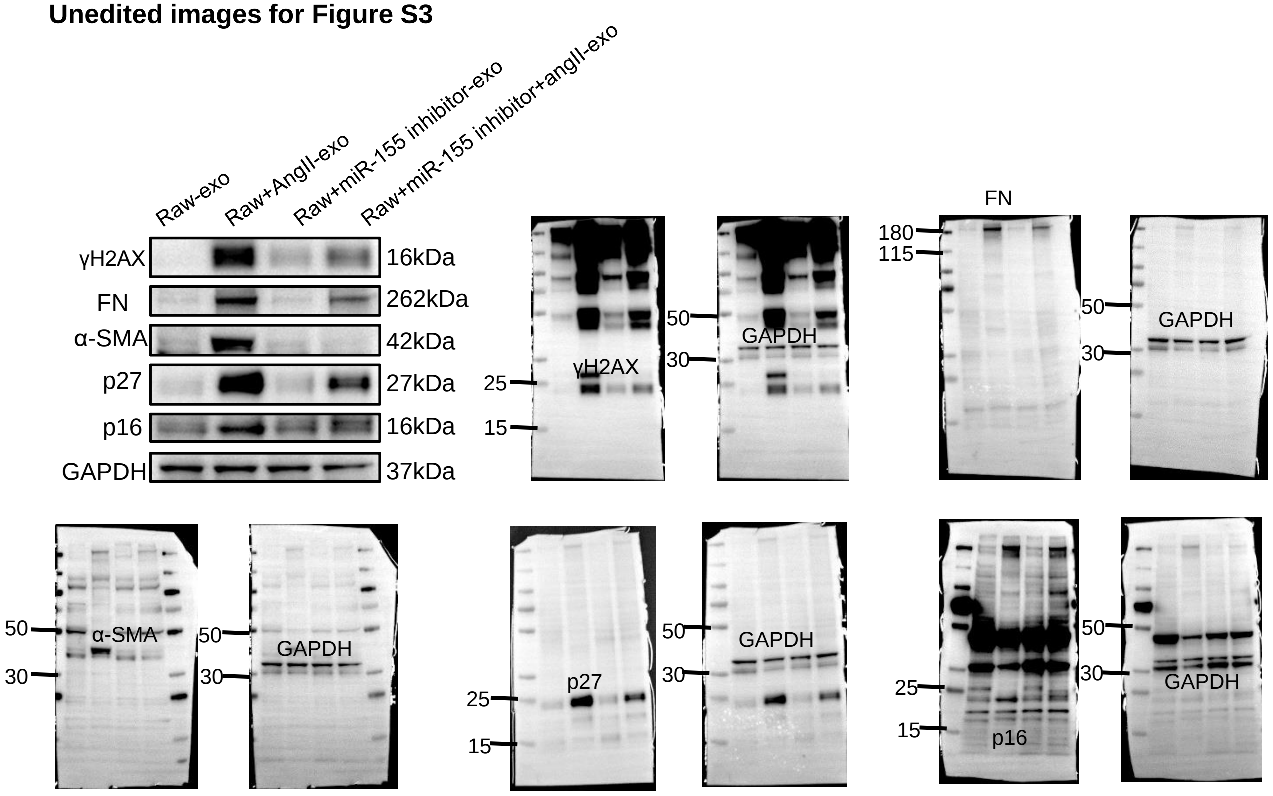

Supplement: Supplementary file 2 — Supplementary Material 2. [file 12964_2024_1708_MOESM2_ESM.pdf]
